# Association of Meibomian Gland Dysfunction with Oral Statin Use

**DOI:** 10.3390/jcm11154632

**Published:** 2022-08-08

**Authors:** Sun-Kyoung Park, Ji-Hye Lee, Ho-Sik Hwang, Hyun-Seung Kim, Kyung-Do Han, Kyung-Sun Na

**Affiliations:** 1 Department of Ophthalmology, College of Medicine, The Catholic University of Korea, Seoul 06591, Korea; 2 Department of Statistics and Actuarial Science, Soongsil University, Seoul 03080, Korea

**Keywords:** meibomian gland dysfunction, dry eye disease, statin, HMG-CoA reductase inhibitor, meiboscore

## Abstract

This retrospective cross-sectional study aimed to determine the association of oral statin use, dry eye disease (DED), and meibomian gland dysfunction (MGD). A total of 93 subjects were included and divided into two groups: statin users (*n* = 45) and nonstatin users (*n* = 47). Significant differences were observed in the total cholesterol (*p* = 0.013), low-density lipoprotein (LDL) (*p* = 0.005), and meiboscore (*p* = 0.000) levels between the two groups. For stratified analysis, the statin group was divided into subgroups according to the type or dose of statin and total duration of statin use. However, there were no differences in clinical features between the subgroups. In multiple regression analysis, meiboscore was significantly associated with age (slope = 0.05, *p* = 0.00) and statin use (slope = −1.19, *p* = 0.00), with an R^2^ of 0.44. Thus, older adults and participants who do not use statin appeared to have higher scores. In conclusion, although the mechanism is unclear, statins may exert a protective effect on the meibomian gland. Further lipidomic studies are required to determine the pharmacological effects of statins on the meibomian gland and other meibum components.

## 1. Introduction

The meibomian gland secretes meibum, which is composed of over 600 types of complex mixture of various polar and non-polar lipids including cholesteryl esters (CEs), triacylglycerol, free cholesterol, free fatty acids (FFAs), phospholipids, wax esters (WEs), and diesters [1,2,3]. The meibum forms a lipid layer in the tear film to stabilize the tear film and protect the ocular surface [4]. Meibomian gland dysfunction (MGD) is defined as “a chronic, diffuse abnormality of the meibomian glands that is commonly characterized by terminal duct obstruction or qualitative or quantitative changes in glandular secretion” [5], which results in evaporative dry eye disease (DED) [6]. MGD is thought to be associated with systemic conditions including aging, sex hormone deficiency, rosacea, and Sjögren’s syndrome [7,8,9,10]. Meanwhile, a limited number of studies have analyzed the relationship between systemic lipid abnormalities and those of the tear film. Although the results are still conflicting and vague, many studies have suggested a possible association between MGD and dyslipidemia [11,12,13,14,15,16,17,18,19].

Dyslipidemia is defined as a disorder of systemic lipid metabolism, characterized by abnormally elevated levels of total blood cholesterol (TC), triglyceride (TG), and low-density lipoprotein (LDL) and/or a reduction in the level of high-density lipoproteins (HDLs) [20]. It is a significant and modifiable risk factor for cardiovascular disease, a major cause of death in adults [21]. It is not yet known whether the use of dyslipidemia medications including 3-hydroxy-3-methylglutaryl-coenzymeA (HMG-CoA) reductase inhibitors, also known as statins, is associated with MGD. Statins, 3-hydroxy-3-methyl-gutaryl coenzyme A reductase inhibitors, are the rate-limiting enzymes in the mevalonate pathway for the biosynthesis of intracellular cholesterol. Statins competitively bind to HMG-CoA reductase, displacing its natural substrate—HMG-CoA. This halts the conversion of HMG-CoA to l-mevalonate, thus ultimately inhibiting cholesterol synthesis [22,23,24]. HMG-CoA reductase expression has been identified in sebaceocytes of the meibomian glands in human eyelid tissue [25]. Oral statins may reduce the increased local cholesterol output of the meibomian glands or accessory glands of Zeis in blepharitis, potentially reducing the burden of MGD [26].

Several clinical and basic studies have postulated that statins may have diabetogenic and anti-inflammatory effects as well as therapeutic effects on nervous system diseases such as cerebral thrombosis or Alzheimer’s disease, coronary heart disease, and cancer [23,24]. However, the effect of statins on meibum composition or meibogenesis has not been investigated. Therefore, a study on the correlation between meibomian gland dysfunction and statins may help establish a new treatment direction for meibomian gland dysfunction. To investigate a potential association between MGD and a history of statin use or dyslipidemia, this cross-sectional study investigated the relationship between dyslipidemia, statins, and MGD.

## 2. Materials and Methods

### 2.1. Patients

This retrospective, cross-sectional study was approved by the Institutional Review Board (IRB) of the Yeouido St. Mary’s Hospital, The Catholic University of Korea (SC22RISI0088) and was conducted in accordance with the ethical principles outlined in the Declaration of Helsinki. Due to the retrospective nature of the study, the IRB of Yeouido St. Mary’s Hospital, The Catholic University of Korea waived the requirement for informed consent. We reviewed the charts of patients who were diagnosed with cataracts and scheduled to undergo cataract surgery from March 2022 to May 2022. Patients included in the study were aged >19 years old. The patients were divided into two groups: (1) statin group, or patients undergoing regular HGM-CoA reductase inhibitor (statin) treatment, and (2) nonstatin group. In the statin group, those who had been taking statins for at least 3 months were included in the study. The exclusion criteria were: age > 80 years, active eye infection, a history of chemical or thermal injury to the ocular surface, previous operation on the eyelid or conjunctiva, hormonal imbalance (especially sex hormones such as postmenopausal hormone therapy or polycystic ovary syndrome), rheumatic conditions (e.g., Sjogren’s syndrome), neurological conditions (e.g., Parkinson’s ds), dermatological diseases (e.g., atopy, rosacea, SJS, psoriasis), history of hematopoietic stem cell transplantation, use of topical steroids or antiglaucoma medications, and use of oral antihistamines, antidepressants, retinoids, or omega-3 fatty acid supplements. The statin group was further divided into subgroups according to the type and dose of statins: (1) atorvastatin (10 and 20 mg), (2) rosuvastatin (5 and 10 mg), and (3) pitavastatin (1, 2, and 4 mg). The statin group was also divided into five subgroups according to the total duration of statin use: (1) <1 year, (2) 1–5 years, (3) 5–10 years, (4) 10–15 years, and (5) >15 years.

### 2.2. Clinical Assessment

Thorough ocular surface examination was conducted on both eyes of all patients with cataracts to find the pre-existing DED and MGD before the cataract surgery, and data from the left eye were used as representatives. The subjects were assessed for ocular surface and meibomian gland findings as well as their reports of subjective symptoms. The following objective tests for MGD were performed in the following order: characterization of DED symptoms using the validated questionnaire Standard Patient Evaluation of Eye Dryness (SPEED), slit-lamp examination of the ocular surface to assess tear break-up time (TBUT), corneal/conjunctival fluorescein staining, meibomian gland expressibility, meibum quality, and noncontact meibography. A 5 min interval or longer was allotted between each test, except between the administration of the SPEED questionnaire and slit-lamp examination.

The SPEED questionnaires was used to grade the level of DED symptomology [27]. The assessment standard of the SPEED questionnaire was derived by summing the scores from the frequency and severity parts of the questionnaire for 3 months. The values of frequency and severity in the SPEED questionnaire were obtained by summing the scores of the eight items (each rated from 0 to 4), and the total SPEED scores ranged from 0 to 28. The results were interpreted as follows: normal eye (score 0) and DED (score 1–28).

Corneal staining was performed using fluorescein sodium-impregnated paper strips (Haag-Sterit, Bern, Switzerland). The strips were wetted with normal saline, and diluted dye was instilled into the ocular surface. After gentle blinking, the degree of corneal staining was graded for five corneal and 2 × 3 conjunctival zones according to the NEI/Industry Schema (range, 0–3 points per zone) [28]. TBUT, the interval between blinking and the first appearance of a dry spot on the tear film, was measured three times consecutively after fluorescein instillation, and the mean value was used.

**Figure 1:**
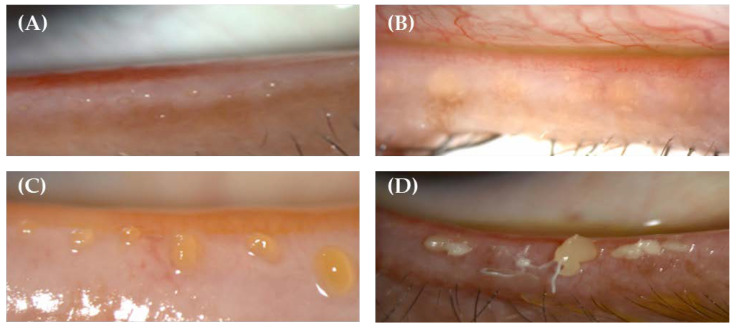
Representative images of meibum quality grading. (**A**) Grade 0, clear fluid; (**B**) Grade 1, cloudy fluid; (**C**) Grade 2, cloudy particulate fluid; (**D**) Grade 3, inspissated, toothpaste-like.

The ability of eight meibomian glands in the central area of the lower eyelid to secrete meibum was tested after applying mechanical pressure using a handheld Meibomian Gland Evaluator™ (TearScience, Morrisville, NC, USA) [29]. The results were scored from 0 to 8 depending on the number of expressible glands found among the eight central glands. Slit-lamp examinations were performed to evaluate the meibum quality, which was assessed in each of the eight glands of the central third of the lower lid on a scale of 0 to 3 for each gland (total score range, 0–24) [30]. Images representative of meibum quality grading are shown in Figure 1A–D.

The thickness of the tear film lipid layer (TFLL), which occupies the most anterior part of the tear film, was measured using a LipiView interferometer (TearScience Inc., Morrisville, NC, USA). The lower eyelids were everted, and meibography images were acquired through non-contact infrared meibography (Lipiview, TearScienceInc., Morrisville, NC, USA). The degree of meibomian gland loss was classified according to the meiboscore described by Arita et al. on a scale of 0–3 as follows: 0, no loss of meibomian gland; 1, area loss < one-third of the total meibomian gland area; 2, area loss between one-third and two-thirds of the total meibomian gland area; and 3, area loss > two-thirds of the total meibomian gland area [31].

Additionally, blood laboratory tests of baseline lipid profiles, including quantification of TC, TG, HDL, and LDL levels, were collected for all patients, as well as ophthalmic evaluations as routine examinations prior to cataract surgery.

### 2.3. Statistical Analysis

Pearson chi-squared and Student’s *t*-tests were used to assess the differences in categorical and continuous variables, respectively, between statin users and non-statin users.

Pearson’s correlation, Student’s t-, and one-way ANOVA tests were employed to test the influences of covariates on DED/MGD parameters, and the finding of a non-significant correlation resulted in no adjustment of DED/MGD parameters for these potentially confounding variables.

In order to identify variables independently associated with variations in DED/MGD parameters, we performed multivariate regression analysis. The model included variables known as DED or MGD risk factors (age, sex, and underlying disease (DM, HTN)) [17,18,19,32,33,34,35,36,37,38], lipid profiles (total cholesterol, TG, LDL, and HDL), and statin use. The variance inflation factor (VIF) was used to check for the problem of multicollinearity among the predictor variables in multiple regression analysis. Any variable with a VIF that exceeded four was excluded from the model, as recommended in the literature; therefore, given that the VIF value of total cholesterol and LDL was 6.782, LDL was excluded from the regression model [36].

Mann–Whitney and one-way ANOVA tests were used to compare the differences in clinical parameters and lipid profile values according to the type or dose of statins and the duration of statin use.

Values are expressed as means and standard deviations. A *p*-value < 0.05 was considered statistically significant. Statistical analyses were performed using Statistical Package for the Social Sciences (SPSS) ver. 22.0 software (IBM Corp., Armonk, NY, USA).

The sample size and power calculations were conducted assuming a type I error of 0.05, type II error of 0.8, and effect size of 0.5. An estimated sample size of 64 participants was obtained for each group. We based our sample size calculation on the limited literature [25,36,38,39].

## 3. Results

### 3.1. Demographics

A total of 92 participants were included in this study. The nonstatin group included 47 participants with a mean age of 67.53 ± 8.39 years (range: 43–80 years), 38.30% of whom were men and 61.70% women. The statin group comprised 45 participants with a mean age of 70.58 ± 5.43 years (range: 59–80 years), 37.78% of whom were men and 62.22% women. There were no statistically significant differences between the two groups in terms of age or sex (*p* = 0.104 and *p* = 0.959, respectively). Additionally, there were no statistically significant differences with regard to underlying diseases (diabetes mellitus and hypertension) between the two groups (*p* = 0.301 and *p* = 0.134, respectively). Demographic characteristics of the participants are presented in Table 1.

### 3.2. Lipid Profiles and Clinical Manifestations of the Statin and Nonstatin Groups

The lipid profiles of the two groups (TC, LDL, TGs, and HDL) and DED or MGD parameters (SPEED, TBUT, corneal stain, conjunctival stain, meibomian gland expressibility, meibum quality, TFLL thickness, and meiboscore) were compared. For the lipid profile, mean TC and LDL levels were significantly lower in the statin group than in the nonstatin group (*p* = 0.0017 and *p* = 0.0055, respectively). There were significant differences in the conjunctival stain scores and meiboscores between the two groups (*p* = 0.027 and *p* = 0.000, respectively). However, the mean SPEED score, TBUT, corneal/conjunctival staining, meibomian gland expressibility, meibum quality, and TFLL thickness were not significantly different between the two groups (Table 1).

### 3.3. Correlation and Comparison between DED/MGD Parameters and Covariates

Pearson’s correlation analysis revealed statistically significant negative correlations between SPEED scores and creatinine levels (r = −0.31, *p* = 0.00) as well as between meibomian gland expressibility and HDL (r = −0.25, *p* = 0.02). There were no significant correlations between the other continuous variables (Table 2).

**Figure 2:**
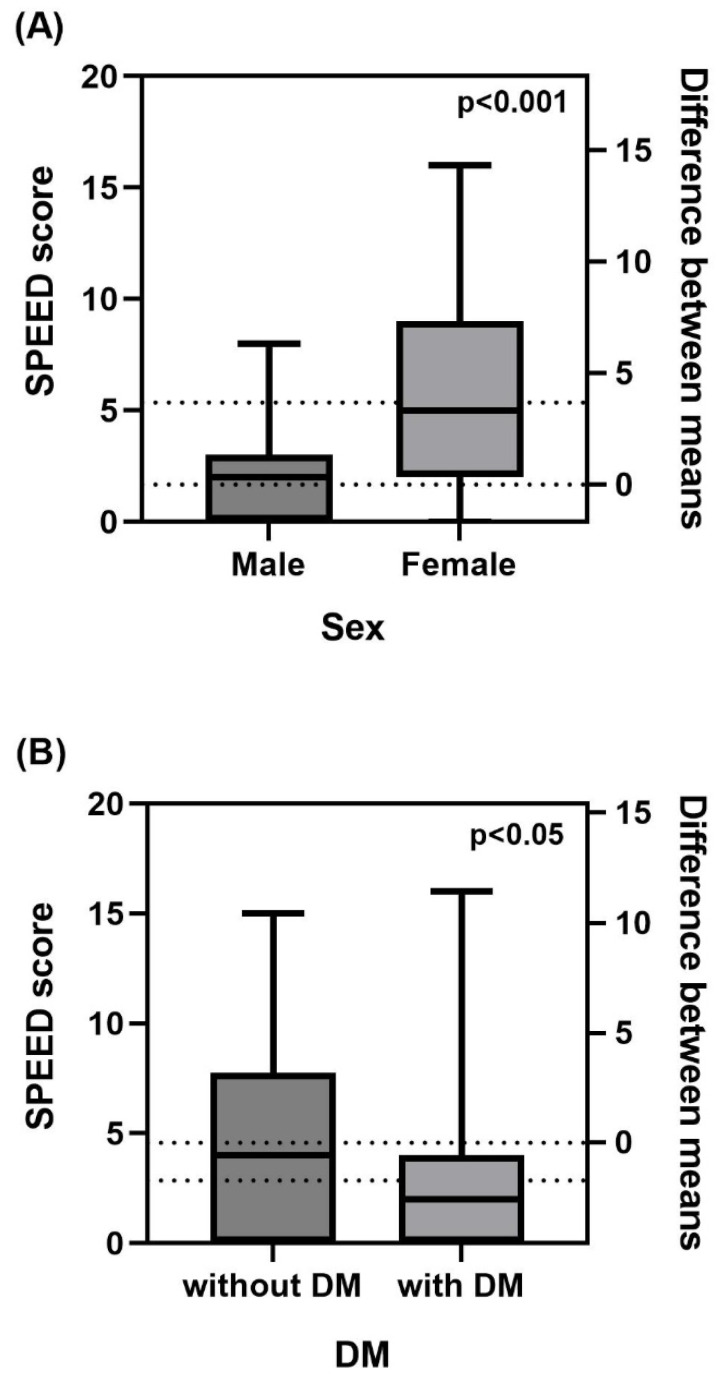

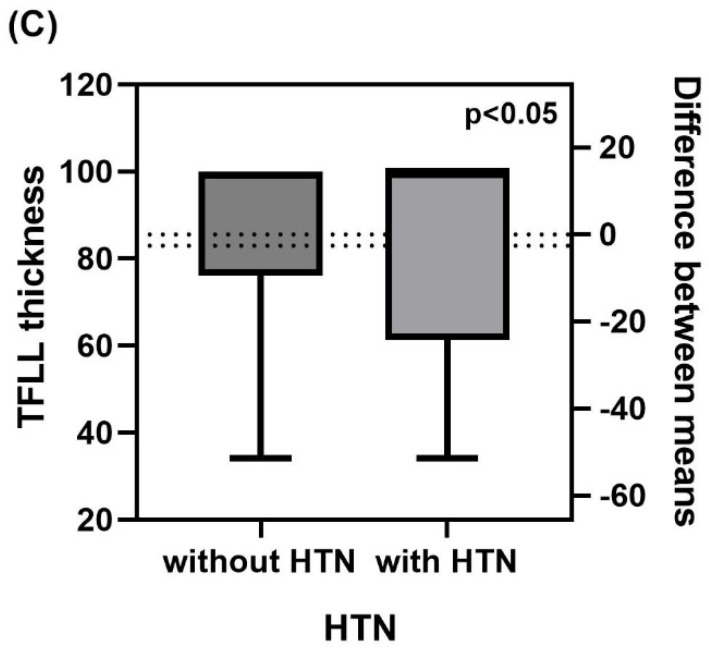
(**A**) SPEED scores in men and women. (**B**) SPEED scores in subjects without DM and those with DM. (**C**) TFLL thickness in subjects without HTN and those with HTN. The median value is indicated by the central horizontal line inside the box, and the lower and upper quartiles by the corresponding horizontal ends of the box. The maximum and minimum values are displayed with vertical lines connecting the points to the center box. The two dotted lines represent the mean value of each, and the distance between the lines represents the difference between the means. In Figure 2C, the median value is “100” for each. SPEED, Standard patient evaluation of eye dryness; DM, diabetic mellitus; HTN, hypertension; TFLL, tear film lipid layer.

Regarding sex, the SPEED score showed a statistically significantly higher mean value in women than in men, and there were no differences between sex for the remaining variables (Figure 2A). For DM and HTN, the SPEED score showed a statistically significant higher mean value in participants without DM than in those with DM (Figure 2B). TFLL thickness in participants with HTN showed significantly higher mean values than in those without HTN (Figure 2C). There were no differences in the remaining variables based on the presence or absence of DM or HTN. In addition, no significant differences were observed between the meiboscores and the covariates.

### 3.4. Associations between DED/MGD Parameters and Covariates

Multiple regression analysis showed a significant association between the meiboscores and age (slope = 0.048, *p* < 0.001) and statin use (slope = −1.187, *p* < 0.001) (R^2^ = 0.44 (Table 3). Thus, meiboscores of older adults and participants who use statins appeared to have 0.048 higher and 1.187 lower scores than those of younger adults and participants who do not use statins, respectively (both *p* < 0.001), when the values of all other confounders, including total cholesterol, were considered to be the same. No associations were found between other clinical parameters (SPEED, TBUT, corneal/conjunctival stain, meibomian gland expressibility, meibum quality, and TFLL thickness) and covariates.

### 3.5. Clinical Manifestations of Statin Subgroups

Supplementary Table S1 shows the clinical manifestations of these statin subgroups. Although rosuvastatin showed the strongest lipid control effect, there were no significant differences in the lipid profiles or DED/MGD parameters among the subgroups. In the atorvastatin and rosuvastatin groups, there were no significant differences in lipid profiles or clinical manifestations according to statin dose (Supplementary Table S2). In addition, no significant differences were observed in the lipid profiles or DED/MGD parameters according to the total duration of statin use (Supplementary Table S3).

## 4. Discussion

Here, we report a retrospective analysis of the association between DED or MGD with statin use. We observed higher meiboscores in the statin group compared with those in the nonstatin group. Moreover, in the multiple regression analysis, age and statin use were significantly associated with meiboscores. Importantly, although there were significant differences in total cholesterol and LDL values between the statin and the nonstatin groups, through multiple regression analysis, it was found that they were not significant meiboscores determinants. Although similar studies have been conducted, the results have been conflicting. A recent study reported that patients treated with statins had a lower risk of developing blepharitis than matched patients without statin treatment [26]. In contrast, based on an administered questionnaire, another recent analysis of data from subjects in the Blue Mountains Eye Study found that patients taking oral statins were more likely to report one or more moderate-to-severe DED symptoms [40]. However, they did not correlate statin use with clinical examination results. The former study determined blepharitis from the diagnosis code for DED, while the latter determined DED from symptoms reported in a questionnaire.

To the best of our knowledge, no previous study has evaluated the association between statin use and DED/MGD parameters in detail. We specifically examined this relationship in this study by dividing the statin group into subgroups according to the type or dose of statin and the total duration of statin use.

It is well-known that lid margin morphologies change with age. They become thicker, more hyperkeratinized, and have more telangiectasia, which may ultimately increase the risk of blepharitis [41]. A recent prospective longitudinal study examined the effect of statins on MG morphological changes over 12 months and revealed a statistically significant increase in total and upper eyelid meiboscores as well as lid margin abnormality scores in the statin group [42]. Even though this study has several strengths such as being a prospective longitudinal study, the duration of observation might have been too short to judge the effect of statins on MG dropout or lid margin abnormality. Although our study was retrospective, we observed the effect of statins administered for less than 1 year to over 15 years.

MGD can be considered a cause of posterior blepharitis and evaporative dry eye, resulting in excess free cholesterol and cholesterol esters in tears [43,44,45,46]. Changes in tear composition disrupt the meibum layer, resulting in inflammatory cell infiltration of the ocular surface epithelium, along with an increase in the expression of proinflammatory cytokines including IL-1β, IL-6, IL-17, IFN-γ, tumor necrosis factor (TNF)-α, and matrix metalloproteinase (MMP)-9 [47,48,49]. Statins and 3-hydroxy-3-methyl-gutaryl coenzyme A reductase inhibitors are rate-limiting enzymes in the mevalonate pathway for the biosynthesis of intracellular cholesterol. They have non-lipid-lowering pleiotropic and HMG-CoA reductase inhibition effects, which are their most important anti-inflammatory and immunomodulatory effects. In an immunohistochemical study by Ooi et al., HMG-CoA reductase expression was found in human eyelid tissue within the sebaceocytes of the meibomian, Zeis, and pilosebaceous glands [25]. An in vitro study by Jameel et al. showed that atorvastatin reduced the production of interleukin (IL)-1, IL-5, IL-6, IL-17, and interferon (IFN)-γ by activated T cells [50]. The protective effect of statins on meibomian gland morphology presented as meibosores can be explained by the anti-inflammatory properties of statins, as well as HMG-CoA reductase inhibition.

In our study, there were no significant associations between statin use and DED or MGD parameters, except for meiboscores. This might be because of the indirect action and relatively low bioavailability of oral statins compared with topical statins. In contrast with our results, an in vivo study that examined the use of topical atorvastatin in patients with DED showed that topical atorvastatin was efficacious for treating DED associated with blepharitis [51]. The authors ascribed that this result might be due to a potentially local, more potent HMG-CoA reductase inhibition on sebaceocytes of meibomian, Zeis, and pilosebaceous glands, as well as through their known anti-inflammatory properties. Lipophilic topical statins may be able to inhibit HMG-CoA reductase locally by penetrating the ocular surface and eyelid ductal structures through their lipid secretions and/or ductal epithelia and sebaceocytes of the acini [39]. Unlike topical statins, oral statins do not act directly on the ocular surface or eyelid ductal structure, resulting in insufficient anti-inflammatory effects in DED or MGD. Moreover, considering the bioavailability of statin therapy, oral medications might have insufficiently affected the HMG-CoA reductase receptor of the tarsal plates in patients with MGD.

Statins have different lipophilicity, potency, and half-life according to their type, as well as different pharmacokinetic profiles, including bioavailability [52]. Previous studies showed that the bioavailability of statins ranges from 5% to 80% depending on the pharmacokinetic properties of the individual statins [22]. Therefore, we examined the association between clinical features and the type or dose of statins as well as the total duration of statin use. However, we did not observe any significant associations between the subgroups. We assumed that these results might be due to the lack of difference in the action intensity of the statins included in the study. According to the 2013 ACC/AHA guidelines, all statins included in the analysis have low or moderate intensities: low intensity (pitavastatin 1 mg daily) and moderate intensity (atorvastatin 10, 20 mg daily, rosuvastatin 5, 10 mg daily, and pitavastatin 2, 4 mg daily). In contrast with our study, a recent retrospective case-control study divided patients with statin use into three categories according to the intensity of action and showed 40% greater odds of a diagnosis of DED in patients on statin regimens of all intensities compared to the nonstatin group [53]. The strength of the previous study is that it is the largest cohort study ever conducted that examined DED in association with statin use. However, misclassification bias and the possibility of under-reporting or over-reporting cannot be excluded since the study defined DED using the diagnosis code for DED and not from clinical examinations as in our study.

The limitations of our study include a relatively small sample size and our inability to observe duration-based associations between statin use and DED or MGD because of the retrospective nature of our study. However, our study differs from previous retrospective studies in that we acquired information about the underlying diseases and history of systemic or topical drug use and performed clinical examinations and blood sampling at the same time as routine examinations prior to cataract surgery. As there were no time intervals between the acquisition of information, clinical examinations, and laboratory tests, the shortcomings of being a retrospective study was somewhat overcome. In addition, this is the first study to analyze the correlation between DED/MGD and the type or dose of statins as well as the total duration of statin use.

Future retrospective studies with a large sample size or long-period observational prospective studies in which the possible confounding factors such as DM, hypertension, stroke, CHD, chalazion, rosacea, Sjögren syndrome, psoriasis, and atopy are thoroughly controlled to elucidate the pathogenic mechanisms involved and to identify any potential therapeutic targets are needed. Additionally, it would be meaningful to analyze whether there are differences in clinical features according to statin intensity, including participants with high-intensity statin use.

A series of studies by Butovich proposed in situ meibogenesis in human tarsal plates [54]. Statins have been shown to disturb the synthesis of sterols and isoprenoids in human meibomian gland epithelial cells via the inhibition of HMG-CoA reductase [25,55,56]. Considering in situ meibogenesis, the alteration of meibum lipid composition by statins may have a negative effect on the meibomian gland. Further lipidomic studies, including the comparison of effects between oral and topical preparations, are required to determine the pharmacological effect of statins on various meibum components.

## 5. Conclusions

Our results suggest that statins may have a protective effect on MG morphology. This may be due to anti-inflammatory properties and HMG-CoA reductase inhibition of statins. This study showed the potential of statins as a new therapeutic agent for DED/MGD. However, there is currently insufficient research to support these results, and the exact mechanism is unknown. Therefore, well-designed clinical and lipidomic studies are needed in the future.

## Figures and Tables

**Table 1:** Demographics and clinical characteristics of participants.

Characteristics	Statin Group	Nonstatin Group	*p*-Value
**Total number of patients**	45	47	
**Age (year)**	70.58 (5.43)	67.53 (8.39)	0.065
**Age (year) range**	59–80	43–80	
**Sex**			0.959
Men (%)	37.78	38.30	
Women (%)	62.22	61.70	
**DM (%)**	53.33	42.55	0.301
**HTN (%)**	37.78	23.40	0.134
**BUN**	16.54 (5.56)	16.33 (5.15)	0.971
**Creatinine**	0.82 (0.23)	0.80 (0.20)	0.250
**AST**	23.43 (6.54)	23.15 (8.08)	0.496
**ALT**	23.05 (10.62)	20.98 (14.18)	0.745
**Total cholesterol (mg/dL)**	157.18 (30.49)	200.58 (48.30)	**0.013**
**LDL (mg/dL)**	79.21 (27.72)	126.64 (41.67)	**0.005**
**Triglyceride (mg/dL)**	141.55 (75.81)	180.04 (171.54)	0.132
**HDL (mg/dL)**	60.08 (13.23)	56.27 (16.12)	0.127
**SPEED**	4.50 (4.50)	3.62 (4.08)	0.497
**TBUT (sec)**	5.05 (3.38)	4.09 (2.06)	0.235
**Corneal stain**	1.20 (1.38)	1.60 (2.15)	0.234
**Conjunctival stain**	3.78 (3.44)	5.67 (4.09)	0.249
**M** **G expressibility**	3.71 (2.53)	3.45 (2.35)	0.547
**Meibum quality**	12.13 (5.17)	13.02 (5.05)	0.904
**TFLL thickness (nm)**	85.80 (19.49)	82.93 (23.11)	0.154
**Meiboscore**			**<0.00** **1**
Grade 0 (%)	78.95	19.51	
Grade 1 (%)	13.16	51.22	
Grade 2 (%)	5.26	21.95	
Grade 3 (%)	2.63	7.32	

Values are presented as the mean (standard deviation) or number (%). DM, diabetes mellitus; HTN, hypertension; LDL, low-density lipoprotein; HDL, high-density lipoprotein; SPEED, Standard Patient Evaluation of Eye Dryness; TUBT, tear film break-up time; MG, meibomian gland; TFLL, tear film lipid layer. The bold stands for “statistically significant”.

**Table 2:** Pearson’s correlations between DED/MGD parameters with continuous covariates.

	**SPEED Score**			**TBUT**			**Corneal Stain**	
	***r***		***p*** **-Value**	***r***	***p*** **-Value**		***r***	***p*** **-Value**
**Age**	−0.101		0.352	0.002	0.983		-0.041	0.701
**BUN**	0.089		0.415	−0.097	0.364		0.129	0.228
**Creatinine**	−0.307		**0.004**	0.125	0.242		−0.028	0.794
**AST**	−0.141		0.197	0.123	0.247		−0.049	0.650
**ALT**	−0.053		0.630	0.054	0.616		0.000	0.998
**Total cholesterol**	0.050		0.661	−0.015	0.895		0.154	0.171
**LDL**	0.024		0.832	−0.021	0.850		0.099	0.382
**Triglyceride**	−0.149		0.192	−0.077	0.493		0.169	0.132
**HDL**	0.215		0.059	0.020	0.856		0.063	0.577
	**Conjunctival Stain**		**M** **G** **Expressibility**		**Meibum Quality**		**TFLL Thickness**	
	***r***	***p*** **-value**	***r***	***p*** **-value**	***r***	***p*** **-value**	***r***	***p*** **-value**
**Age**	−0.210	0.050	−0.090	0.392	−0.004	0.968	0.077	0.484
**BUN**	−0.041	0.704	0.068	0.523	−0.080	0.448	0.185	0.092
**Creatinine**	−0.068	0.530	0.062	0.557	−0.074	0.484	0.084	0.449
**AST**	−0.066	0.544	−0.131	0.216	0.171	0.104	−0.143	0.193
**ALT**	−0.075	0.490	−0.051	0.631	0.095	0.373	−0.173	0.116
**Total cholesterol**	0.138	0.225	−0.047	0.670	−0.047	0.675	−0.027	0.814
**LDL**	0.151	0.183	−0.005	0.968	−0.029	0.794	−0.022	0.845
**Triglyceride**	0.030	0.791	0.033	0.769	−0.107	0.337	0.101	0.377
**HDL**	0.090	0.428	−0.250	**0.022**	0.088	0.430	−0.130	0.256

SPEED, Standard Patient Evaluation of Eye Dryness; TBUT, tear film break-up time; MG, meibomian gland; TFLL, tear film lipid layer; BUN, blood urea nitrogen; AST, aspartate transaminase; ALT, alanine transferase; LDL, low-density lipoprotein; HDL, high-density lipoprotein. The bold stands for “statistically significant”.

**Table 3:** A multiple regression analysis evaluating meiboscores as dependent variable.

Dependent Variable	Independent Variable *	Slope	SE	*p*-Value	R^2^
**Meiboscore**	**Age**	0.048	0.013	**<0.001**	0.441
	**Sex**	−0.206	0.195	0.295	
	**DM**	0.293	0.172	0.094	
	**HTN**	0.073	0.208	0.727	
	**Total cholesterol**	−0.004	0.003	0.111	
	**Triglyceride**	0.001	0.001	0.225	
	**HDL**	0.011	0.007	0.093	
	**Statin use**	−1.187	0.195	**<0.001**	

HDL, high-density lipoprotein; DM, diabetes mellitus; HTN, hypertension. The bold stands for “statistically significant”. * Note: low-density lipoprotein was excluded from the analysis because of its collinearity with total cholesterol.

## Data Availability

Data are available on request from corresponding authors.

## Supplementary Materials

The following supporting information can be downloaded at: https://www.mdpi.com/article/10.3390/jcm11154632/s1, Table S1: Comparison of serum lipid profile values and clinical parameters by statin type; Table S2: Comparison of serum lipid profile values and clinical parameters according to statin dose; Table S3: Comparison of serum lipid profile values and clinical parameters according to total duration of statin use.
