# Supplementary material for: Association of Meibomian Gland Dysfunction with Oral Statin Use
Source: J Clin Med. 2022 Aug 8;11(15):4632. doi: 10.3390/jcm11154632 (PMC9369571; doi:10.3390/jcm11154632)
Supplement: Supplementary file 1 [file jcm-11-04632-s001.zip › jcm-1818607-supplementary.pdf]

Supplementary Table S1. Comparison of serum lipid profile values and clinical parameters by statin type

	**Atorvastatin (n=16)**	**Rosuvastatin (n=16)**	**Pitavastatin (n=11)**	***p-value***
**Total cholesterol (mg/dL)**	153.47 (38.92)	151.75 (19.30)	161.556 (25.075)	0.439
**LDL (mg/dL)**	78.67 (34.18)	71.75 (21.98)	82.556 (21.477)	0.468
**Triglyceride (mg/dL)**	147.40 (80.43)	113.00 (40.30)	162.111 (97.257)	0.475
**HDL (mg/dL)**	56.53 (11.26)	65.33 (16.54)	57.333 (9.552)	0.164
**SPEED**	3.40 (3.18)	5.33 (5.37)	4.900 (5.405)	0.760
**TBUT (sec)**	4.76 (2.23)	5.85 (4.90)	4.414 (1.985)	0.832
**Corneal stain**	0.88 (1.26)	1.38 (1.45)	1.091 (1.300)	0.532
**Conjunctival stain**	3.44 (2.76)	3.25 (3.17)	4.182 (4.513)	0.918
**MG expressibility**	4.31 (2.24)	3.25 (2.38)	3.636 (3.295)	0.387
**MG quality**	11.25 (4.67)	12.69 (4.30)	12.636 (7.298)	0.687
**TFLL thikness(nm)**	93.94 (9.43)	75.57 (26.01)	88.000 (15.240)	0.164
**Meiboscore**				0.404
Grade 0 (%)	80.00	84.62	75	
Grade 1 (%)	13.33	15.38	0	
Grade 2 (%)	6.67	0.00	12.5	
Grade 3 (%)	0.00	0.00	12.5	

Values are presented as the mean (standard deviation) or number (%). LDL low-density lipoprotein, HDL high-density lipoprotein, SPEED Standard patient evaluation of eye dryness, TUBT tear film break-up time, MG meibomian gland, TFLL tear film lipid layer. The bolds stand out for the "statistically significant".

Supplementary Table S2. (A) Comparison of serum lipid profile values and clinical parameters according to atorvastatin dose **(B)** Comparison of serum lipid profile values and clinical parameters according to

rosuvastatin dose.

Table S2(A)

	Atorvastatin		*p-value*
	**10mg**	**20mg**	
**Total cholesterol (mg/dL)**	155.42 (41.05)	145.67 (34.93)	0.470
**LDL (mg/dL)**	80.50 (37.43)	71.33 (19.30)	0.885
**Triglyceride (mg/dL)**	146.50 (65.12)	151.00 (148.09)	0.564
**HDL (mg/dL)**	56.67 (12.17)	56.00 (8.54)	0.942
**SPEED**	2.83 (3.21)	5.67 (2.08)	0.160
**TBUT (sec)**	4.66 (2.47)	5.20 (0.36)	0.459
**Corneal stain**	1.08 (1.32)	0.00 (0.00)	0.118
**Conjunctival stain**	3.62 (2.90)	2.67 (2.31)	0.838
**MG expressibility**	4.46 (2.44)	3.67 (1.15)	0.337
**MG quality**	10.77 (4.73)	13.33 (4.62)	0.491
**TFLL thikness(nm)**	93.46 (10.10)	96.00 (6.93)	0.757
**Meiboscore**			0.519
Grade 0 (%)	83.33	66.67	
Grade 1 (%)	8.33	33.33	
Grade 2 (%)	8.33	0.00	
Grade 3 (%)	0.00	0.00	

Table S2(B)

	Rosuvastatin		*p-value*
	**5mg**	**10mg**	

**Total cholesterol (mg/dL)**	157.80 (24.05)	147.43 (15.66)	0.164
**LDL (mg/dL)**	83.20 (29.22)	63.57 (11.37)	0.290
**Triglyceride (mg/dL)**	111.80 (43.29)	113.86 (41.55)	0.807
**HDL (mg/dL)**	61.80 (21.90)	67.86 (12.80)	0.744
**SPEED**	5.14 (5.52)	5.50 (5.61)	0.907
**TBUT (sec)**	7.43 (7.10)	4.62 (1.84)	0.874
**Corneal stain**	1.43 (1.81)	1.33 (1.22)	0.826
**Conjunctival stain**	2.57 (3.60)	3.78 (2.91)	0.328
**MG expressibility**	3.86 (2.27)	2.78 (2.49)	0.335
**MG quality**	11.29 (4.39)	13.78 (4.15)	0.415
**TFLL thickness(nm)**	82.67 (24.88)	70.25 (27.18)	0.543
**Meiboscore**			0.155
Grade 0 (%)	100.00	71.43	
Grade 1 (%)	0.00	28.57	
Grade 2 (%)	0.00	0.00	
Grade 3 (%)	0.00	0.00	

Values are presented as mean (standard deviation) or number (%). LDL Low-density lipoprotein, HDL high-density lipoprotein, SPEED Standard patient evaluation of eye dryness, TUBT tear film break-up time, MG meibomian gland, TFLL tear film lipid layer. The bolds stand out for the "statistically significant".

Supplementary Table S3. Comparison of serum lipid profile values and clinical parameters according to total duration of statin use.

	**Duration**				***p-value***
	**<5years (n=16)**	**5-9years (n=8)**	**10-14years (n=9)**	**≥ 15years (n=10)**	

**Total cholesterol (mg/dL)**	168.86 (27.43)	166.17 (35.10)	142.22 (34.62)	148.00 (21.52)	0.147
**LDL (mg/dL)**	87.86 (29.38)	88.83 (35.59)	68.00 (24.99)	70.56 (17.56)	0.240
**Triglyceride (mg/dL)**	153.07 (88.69)	120.50 (40.31)	179.89 (86.31)	99.33 (33.35)	0.135
**HDL (mg/dL)**	60.00 (16.59)	61.50 (10.97)	53.78 (7.21)	65.56 (12.47)	0.213
**SPEED**	4.40 (5.18)	5.89 (5.04)	4.70 (4.14)	2.88 (2.90)	0.542
**TBUT (sec)**	5.37 (2.47)	4.43 (1.80)	4.15 (2.14)	6.03 (5.94)	0.447
**Corneal stain**	1.25 (1.39)	1.11 (1.36)	1.30 (1.16)	1.10 (1.73)	0.864
**Conjunctival stain**	2.81 (3.41)	4.67 (3.57)	3.90 (3.07)	4.40 (3.86)	0.431
**MG expressibility**	3.94 (2.95)	4.00 (2.45)	4.00 (2.31)	2.80 (2.20)	0.614
**MG quality**	12.81 (5.36)	11.44 (5.27)	10.10 (5.11)	13.70 (4.83)	0.393
**TFLL thikness(nm)**	80.25 (18.57)	91.89 (10.46)	92.20 (18.50)	80.60 (25.94)	0.283
**Meiboscore**					0.154
Grade 0 (%)	81.82	100.00	50.00	87.50	
Grade 1 (%)	9.09	0.00	30.00	12.50	
Grade 2 (%)	0.00	0.00	20.00	0.00	
Grade 3 (%)	9.09	0.00	0.00	0.00	

Values are presented as the mean (standard deviation) or number (%). LDL low-density lipoprotein, HDL high-density lipoprotein, SPEED Standard patient evaluation of eye dryness, TUBT tear film break-up time, MG meibomian gland, TFLL tear film lipid layer. The bolds stand out for the “statistically significant”.
